# Proteomic Analysis of *Streptococcus suis* During Exposure to Intracellular Condition of Human Macrophage U937 Cells

**DOI:** 10.3390/ijms27010128

**Published:** 2025-12-22

**Authors:** Peerarin Prangsuwan, Orathai Yinsai, Sittiruk Roytrakul, Kwanjit Duangsonk

**Affiliations:** 1 Regional Medical Science Center 12 Songkhla, Songkhla 90100, Thailand; prangsuwan.p@gmail.com; 2 Office of Research Administration, Chiang Mai University, Chiang Mai 50200, Thailand; orathai.yinsai@gmail.com; 3 Department of Microbiology, Faculty of Medicine, Chiang Mai University, Chiang Mai 50200, Thailand; 4 Functional Proteomics Technology Laboratory, National Center for Genetic Engineering and Biotechnology, National Science and Technology for Development Agency, Pathum Thani 12120, Thailand; sittiruk@biotec.or.th

**Keywords:** *Streptococcus suis*, proteomic analysis, invasion, macrophage

## Abstract

*Streptococcus suis* is an important zoonotic pathogen responsible for severe infections in pigs and humans. Its capacity to survive within phagocytic cells is considered a key virulence mechanism that contributes to dissemination and persistence in host tissues. This study employed comparative proteomic profiling to investigate intracellular adaptation of *S. suis* serotypes 2 (SS2) and 14 (SS14) during infection of human U937 macrophages. Five isolates originating from humans and pigs were analyzed using gel electrophoresis with liquid chromatography–tandem mass spectrometry (GeLC–MS/MS), revealing 118 differentially expressed proteins grouped into 11 functional categories. Translation-related proteins represented the largest group (48%), including upregulated ribosomal subunits (30S: S2, S5, S7, S8, S12, S15; 50S: L1, L5, L18, L22, L24, L33, L35) and translation factors such as GidA/TrmFO and RimP. Enrichment of carbohydrate metabolism and DNA replication proteins, including phosphoenolpyruvate carboxylase (PEP), UDP-N-acetylglucosamine pyrophosphorylase (GlmU), and ATP-dependent DNA helicase RuvB, indicated metabolic reprogramming and stress adaptation under intracellular conditions. Stress-response proteins such as molecular chaperone DnaK were also induced, supporting their multifunctional, “moonlighting” roles in virulence and host interaction. Comparative analysis showed that SS2 expressed a broader range of adaptive proteins than SS14, consistent with its higher virulence potential. These findings reveal conserved intracellular responses centered on translation, energy metabolism, and stress tolerance, which enable *S. suis* to survive within human macrophages. Integration of these intracellular proteomic signatures with previous exoproteomic, peptidomic, and network-based studies highlights translational and metabolic proteins—particularly DnaK, enolase, elongation factor EF-Tu, and GlmU—as multifunctional candidates linking survival and immunogenicity. This work establishes a comparative proteomic foundation for understanding *S. suis* intracellular adaptation and highlights potential targets for future vaccine or therapeutic development against this zoonotic pathogen.

## 1. Introduction

*Streptococcus suis* is a major zoonotic pathogen and a significant cause of infectious diseases in pigs worldwide [1]. Traditionally classified into 35 serotypes (1–34 and 1/2) based on capsular polysaccharides [2], recent taxonomic revisions reassigned serotypes 20, 22, 26, 32, and 33 to other species [3], leaving 29 recognized serotypes. Among these, serotype 2 (SS2) is most frequently isolated from pigs and humans, with serotypes 1, 4, 5, 7, 9, 14, 16, and 24 also associated with disease [1]. *S. suis* commonly colonizes the upper respiratory tract of healthy pigs [1,4], but in piglets, it can cause septicemia, meningitis, endocarditis, and arthritis [5]. Human infection typically follows contact with pigs or consumption of raw pork and manifests as meningitis, septicemia, or streptococcal toxic-shock-like syndrome [6,7].

Pathogenesis involves adherence to and invasion of epithelial cells, survival in phagocytes, and dissemination via a “Trojan horse” mechanism [8,9]. *S. suis* crosses the blood–brain barrier primarily through direct adhesion to and invasion of brain microvascular endothelial cells [10]. Virulence factors such as the cholesterol-dependent cytolysin suilysin contribute to this process by disrupting endothelial integrity [11]. Serotype 2 exhibits cytotoxicity toward macrophages, contributing to bacteremia and septicemia [12]. Epidemiologically, SS2 predominates in human cases across Asia, though serotypes 14, 9, and 1/2 are occasionally reported [1,4,13,14,15]. In Thailand, SS2 is most common in human isolates, whereas SS14 is more frequently recovered from pigs [13,14,15]. These patterns highlight the need for region-specific surveillance, public health measures, and serotype-targeted vaccines.

Proteomic analyses provide insight into bacterial adaptation to host environments [16]. Previous studies in SS2 revealed that virulent strains upregulate ribosomal proteins (S2, S5, S12, L1, L33), translation factors (EF-Tu, EF-G, EF-4), metabolic enzymes (PEP carboxylase, galactokinase), and stress-response proteins (DnaK, GlmU), supporting intracellular survival, metabolic flexibility, and virulence [17,18,19,20,21]. Enolase, a moonlighting glycolytic enzyme, enhances blood-brain barrier permeability and immune evasion [22,23]. Secretome and surface proteome studies also identified adhesins and toxins such as suilysin that mediate host colonization and cytotoxicity [23,24].

Although transcriptomic studies have revealed niche-specific gene regulation in *S. suis* [25], the protein-level response during macrophage infection remains unexplored. Proteomic approaches can reveal post-transcriptional adaptations—such as metabolic reprogramming, stress response induction, and virulence factor regulation—that are critical for intracellular survival [26]. Therefore, the objective of this study was to perform a comparative proteomic analysis of intracellular *S. suis* (SS2 and SS14) from human and porcine origins during macrophage contact, aiming to identify conserved and strain-specific protein adaptations that may underlie survival and virulence.

Despite these findings, proteomic studies are largely limited to SS2, in vitro culture, or non-Thai isolates. Data on SS14, human-derived Southeast Asian strains, and intracellular adaptations within macrophages are scarce. Here, we use LC-MS/MS to profile intracellular SS2 and SS14 recovered from human U937 macrophages, comparing them to culture-grown bacteria. By identifying proteins differentially expressed during intracellular survival, including ribosomal proteins, translation factors, metabolic enzymes, and stress-response proteins, we aim to elucidate mechanisms underlying host adaptation, survival, and dissemination. Insights from this study may provide mechanistic evidence to support the development of targeted therapeutic and preventive strategies, including vaccine candidates, against *S. suis* infections in pigs and humans.

## 2. Results

### 2.1. Efficient Intracellular Invasion of Human Macrophages by S. suis

**Figure 1:**
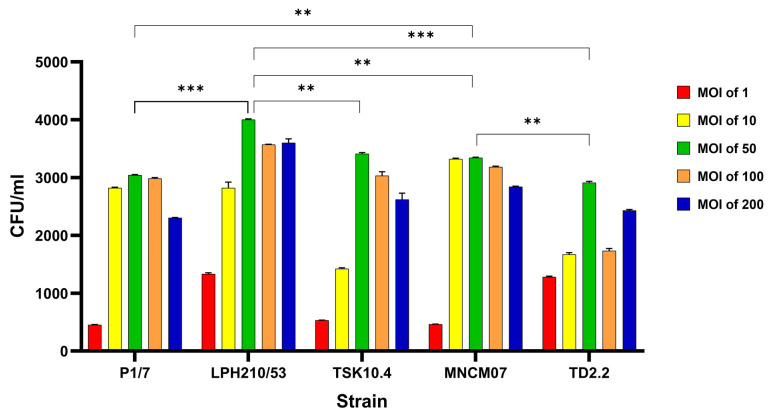
Invasion of U937 macrophage cells by *S. suis* SS2 strains (P1/7, LPH210/53, TSK10.4) and SS14 strains (MNCM07, TD2.2) at MOIs of 1, 10, 50, 100, and 200. Bars represent mean CFU/mL, and error bars indicate standard deviations. Statistical comparisons among strains at each MOI were performed using two-way ANOVA. Asterisks denote statistical significance: ** *p* < 0.01, *** *p* < 0.001.

To optimize conditions for the intracellular proteomic analysis, we first evaluated the invasion efficiency of *S. suis* in U937 macrophages using multiplicities of infection (MOIs) of 1, 10, 50, 100, and 200 (Figure 1). Among the conditions tested, an MOI of 50 yielded the highest intracellular bacterial load. This pattern aligns with the well-described intermediate-MOI optimum in macrophage infection models, where bacterial internalization is maximized without inducing the cytotoxic host–cell responses typically triggered at very high multiplicities of infection. This MOI was therefore selected for subsequent proteomic experiments.

All five *S. suis* isolates—representing SS2 and SS14 from both human and pig origins—were able to invade U937 cells, confirming that human macrophages are highly permissive to *S. suis* entry. However, significant strain-dependent differences were observed. The clinical isolates LPH210/53 (SS2) and MNCM07 (SS14) exhibited significantly higher intracellular bacterial counts across all MOIs compared with the pig-derived strains P1/7 (SS2), TSK10.4 (SS2), and TD2.2 (SS14). These results suggest that clinical isolates may possess a greater capacity for macrophage invasion compared with animal-derived strains, which could be related to differences in their pathogenic behavior.

### 2.2. Global Proteomic Overview of Intracellular S. suis

**Figure 2:**
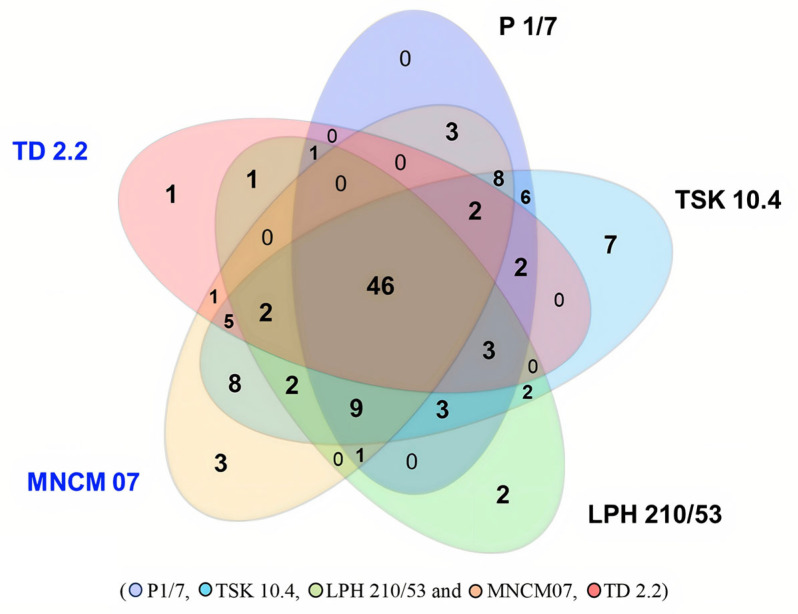
Comparative proteomic profiling of *S. suis* serotypes during macrophage exposure. The Venn diagrams illustrate the number of differentially expressed proteins (DEPs) identified in *S. suis* serotype 2 (P1/7, TSK 10.4, LPH 210/53) and serotype 14 (MNCM07, TD 2.2) following exposure to human macrophages.

Protein profiles of *S. suis* during exposure to human macrophages (U937) were analyzed by SDS-PAGE. Protein bands were fractionated into 15 molecular weight ranges prior to digestion and LC-MS/MS analysis. A total of 118 proteins were detected, and differential expression analysis revealed strain-specific up-regulation during macrophage exposure (Figure 2).

**Figure 3:**
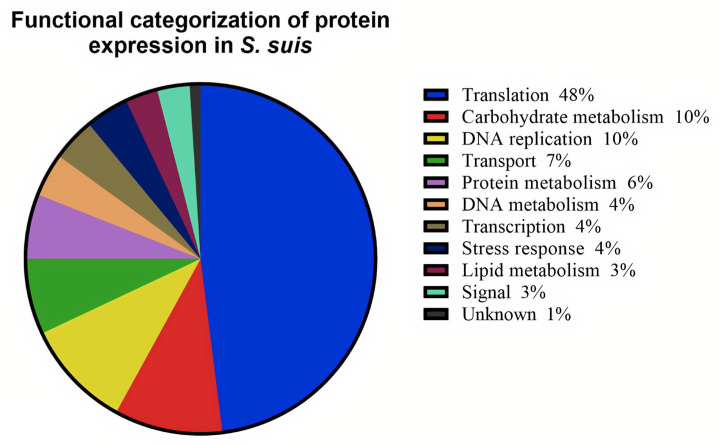
Functional categorization of differentially expressed proteins of *S. suis* after exposure to human macrophages (U937).

The majority of up-regulated proteins were involved in translation (48%), followed by carbohydrate metabolism (10%), DNA replication (10%), transport (7%), protein metabolism (6%), DNA metabolism (4%), transcription (4%), stress response (4%), lipid metabolism (3%), signal transduction (3%), and proteins of unknown function (1%) (Figure 3).

### 2.3. Upregulation of Translation Machinery During Intracellular Adaptation

Strain-specific comparisons of protein expression revealed unique up-regulation patterns. In TSK 10.4, seven proteins were exclusively up-regulated, including keto-acid reductoisomerase, phosphate acyltransferase, chorismate synthase, 30S ribosomal proteins S7 and S8, UPF0348 protein SSU98_0368, and elongation factor 4 (Table S1). LPH 210/53 exhibited specific up-regulation of aspartate-tRNA ligase and ATP-dependent helicase/nuclease subunit A (Table S2), while MNCM 07 uniquely expressed tRNA threonylcarbamoyladenosine biosynthesis protein Gcp, queuine tRNA-ribosyltransferase, and purine nucleoside phosphorylase DeoD-type (Table S3). TD 2.2 specifically up-regulated 50S ribosomal protein L2 (Table S4).

At the serotype level, three proteins—alanine-tRNA ligase, 50S ribosomal protein L1, and UDP-N-acetylglucosamine pyrophosphorylase—were consistently up-regulated in all SS2 isolates (Table S5), whereas only 30S ribosomal protein S5 was up-regulated in SS14 isolates (Table S6). Analysis by isolate origin showed that 50S ribosomal protein L33 and enolase were up-regulated in pig isolates, while no proteins were commonly up-regulated across clinical isolates (Table S7). Most up-regulated proteins were associated with translation and metabolic processes.

Across all five strains of SS2 and SS14, 46 proteins were similarly expressed, including key enzymes and components of protein synthesis such as 6-phosphofructokinase, phosphopentomutase, DNA polymerase III PolC-type, dihydroxy-acid dehydratase, GTPase Era, DnaK, glycine-tRNA ligase β subunit, argininosuccinate lyase, 50S ribosomal protein L18, peptidyl-tRNA hydrolase, and SecA (Table S8).

**Figure 4:**
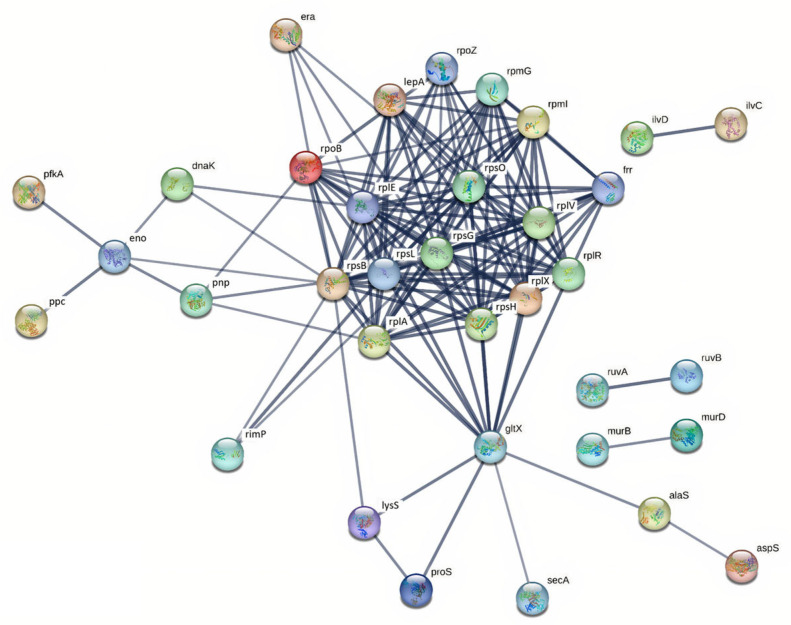
Protein–protein interaction network of up-regulated proteins in *S. suis* SS2 isolates during exposure to human macrophages. The network was analyzed by STRING with a confidence score ≥ 0.7. The nodes represent up-regulated proteins. The lines represent the predicted functional associations. The line thickness means level of the interactions between proteins. Proteins without interactions were excluded from the network visualization.

**Figure 5:**
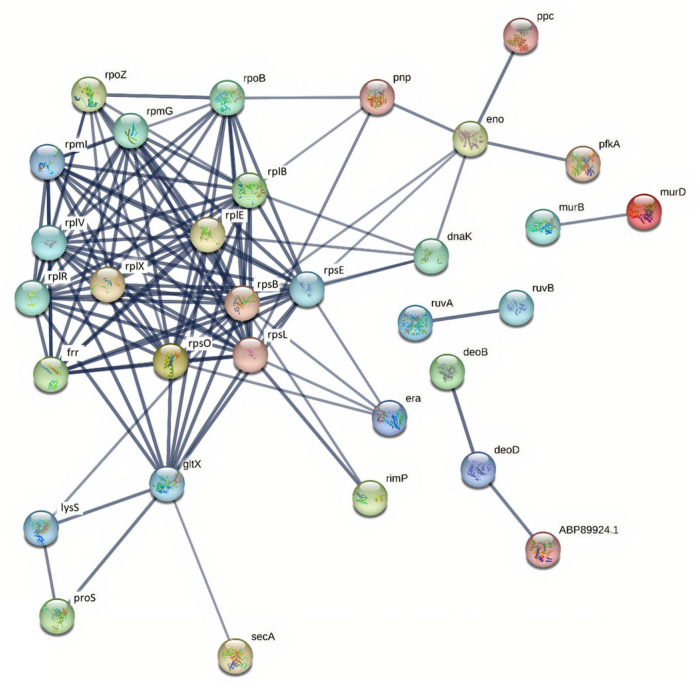
Protein–protein interaction network of up-regulated proteins in *S. suis* SS14 isolates during exposure to human macrophages. The network was analyzed by STRING with a confidence score ≥ 0.7. The nodes represent up-regulated proteins. The lines represent the predicted functional associations. The thick lines mean strong interactions between proteins. Proteins without interactions were excluded from the network visualization.

Overall, SS2 isolates from patients and pigs exhibited 51 and 58 up-regulated proteins, respectively, whereas SS14 isolates showed 50 up-regulated proteins regardless of origin. Protein interaction networks for SS2 and SS14 isolates are illustrated in Figure 4 and Figure 5. Comparatively, SS2 displayed a higher number of up-regulated proteins than SS14, with pig-derived SS2 isolates showing the greatest up-regulation, whereas SS14 showed no significant difference between isolate origins (Table 1).

### 2.4. Metabolic Reprogramming Supports Intracellular Survival

Protein–protein interaction (PPI) networks of up-regulated proteins in *S. suis* SS2 and SS14 were analyzed using the STRING web-based tool. In SS2, 35 proteins formed a connected network, predominantly associated with translation, transcription, and carbohydrate metabolism (Figure 4). Ribosomal proteins were identified as key hubs within the network. Similarly, 35 proteins in SS14 formed a connected network with comparable functional associations and major hub proteins (Figure 5).

### 2.5. Activation of DNA Repair and Stress-Response Pathways

The expression patterns of 118 proteins in SS2 and SS14 during macrophage exposure were analyzed using Multi-Experiment Viewer (MeV). Proteins related to translation were the largest group (57 proteins), followed by carbohydrate metabolism (12 proteins) and DNA replication (12 proteins).

**Figure 6:**
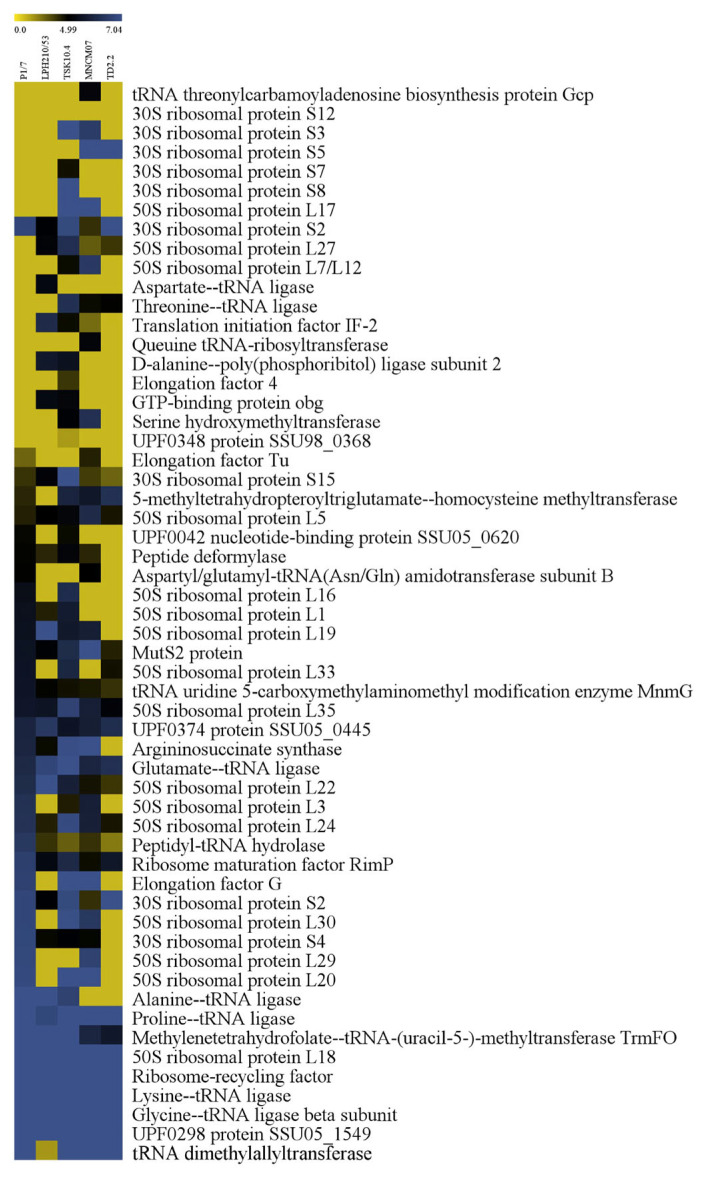
Heat map representation of the translation-related proteins of 5 *S. suis* strains; P1/7, LPH 210/53, TSK 10.4, MNCM 07 and TD 2.2. Level of protein expression is marked using a color scale ranging from yellow (no change), dark (a little) and blue (the highest) up-regulation ratio.

Within the translation group, 16 proteins were consistently expressed across all strains, including 30S ribosomal protein S15, 50S ribosomal protein L5, MutS2, UPF0374 protein SSU05_0445, glutamate-tRNA ligase, lysine-tRNA ligase, and ribosomal recycling factor. Strain-specific proteins included tRNA threonylcarbamoyladenosine biosynthesis protein Gcp and queuine tRNA-ribosyltransferase in MNCM07, aspartate-tRNA ligase in LPH 210/53, and 30S ribosomal protein S7 and 50S ribosomal protein L2 in TSK 10.4 and TD 2.2, respectively (Figure 6).

**Figure 7:**
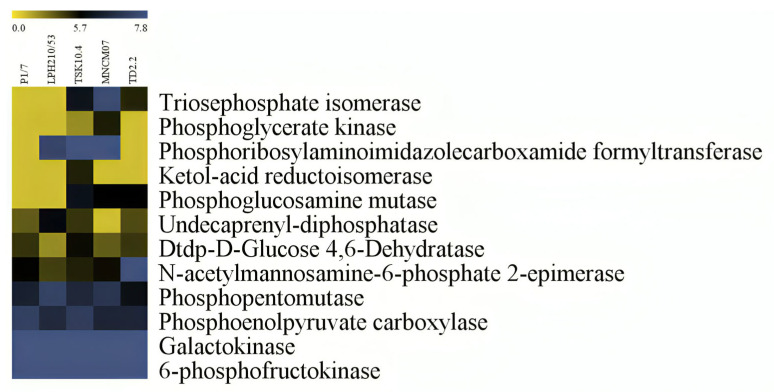
Heat map representation of the carbohydrate metabolism-related proteins of 5 *S. suis* strains; P1/7, LPH 210/53, TSK 10.4, MNCM 07 and TD 2.2. Level of protein expression is marked using a color scale ranging from yellow (no change), dark (a little) and blue (the highest) up-regulation ratio.

In the carbohydrate metabolism group, five proteins were similarly expressed in all strains, including phosphopentomutase, galactokinase, 6-phosphofructokinase, and phosphoenolpyruvate carboxylase. Keto-acid reductoisomerase was specifically expressed in TSK 10.4 (Figure 7).

**Figure 8:**
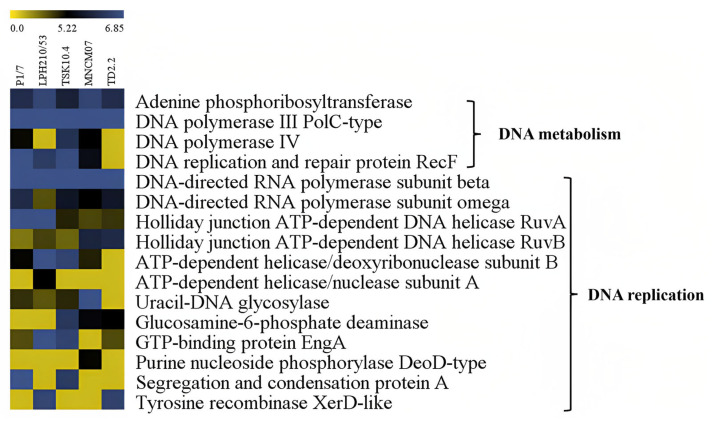
Heat map representation of the DNA metabolism and DNA replication-related proteins of *S. suis* five strains; P1/7, LPH 210/53, TSK 10.4, MNCM 07 and TD 2.2. Level of protein expression is marked using a color scale ranging from yellow (no change), dark (a little) and blue (the highest) up-regulation ratio.

Among DNA replication proteins, four were expressed across all strains—DNA polymerase III PolC-type, DNA-directed RNA polymerase subunit β, DNA-directed RNA polymerase subunit ω, and Holliday junction ATP-dependent DNA helicase RuvA—while ATP-dependent helicase/nuclease subunit A was specific to LPH 210/53 (Figure 8).

## 3. Discussion

*Streptococcus suis* is a significant pathogen in swine and a zoonotic agent in humans, causing a wide range of pathological conditions such as arthritis, endocarditis, meningitis, and septicemia in pigs, as well as streptococcal toxic shock syndrome (STSS), meningitis, and permanent hearing loss in humans [1,6,27]. The pathogen poses a major health risk for individuals in close contact with pigs or pork products and represents a leading cause of sudden death in pigs [28]. Its zoonotic potential underscores the urgent need to identify virulence-associated and infection-related proteins to inform effective control strategies [24].

In this study, comparative proteomic profiling of *S. suis* serotype 2 (SS2; three strains) and serotype 14 (SS14; two strains) was conducted using isolates from a diseased pig (SS2: P1/7, reference strain), healthy pigs (SS2: TSK 10.4; SS14: TD 2.2), and human patients (SS2: LPH 210/53; SS14: MNCM 07). All strains efficiently invaded human macrophage U937 cells at an optimal MOI of 50, yielding high intracellular bacterial counts.

The observation that an MOI of 50 produced the highest recoverable intracellular CFU is consistent with classical macrophage infection dynamics [29]. While low MOIs limit bacterial uptake, excessively high MOIs can induce macrophage stress, cytotoxicity, or programmed cell death—phenomena documented for pathogens like *Staphylococcus aureus* and *Salmonella*—which ultimately reduce the recovery of viable intracellular bacteria [30,31,32]. Thus, MOI 50 likely represents an optimal balance between efficient phagocytosis and preserved host–cell integrity for bacterial recovery.

This observation confirms prior findings that human macrophages are highly susceptible to *S. suis* invasion and supports the concept that intracellular survival within phagocytes is a crucial virulence strategy [27].

Proteomic analysis identified 118 proteins spanning 11 functional categories, with translation-related proteins representing the largest group (48%). Highly upregulated ribosomal proteins included 30S subunits S2, S5, S7, S8, S12, S15 and 50S subunits L1, L5, L18, L22, L24, L33, and L35. Previous studies have reported similar upregulation of ribosomal proteins in virulent SS2 strains under host-simulated environments, such as growth in blood-containing media or during host infection [33,34]. For instance, 30S ribosomal protein S2 is known to influence translational fidelity and bacterial virulence, consistent with findings in *Escherichia coli*, where single amino acid substitutions in ribosomal proteins significantly affected translation efficiency and bacterial fitness [35]. The upregulation of S5 in SS14 isolates mirrors these observations and suggests serotype-specific modulation of translational machinery.

Translation factors such as glycine-tRNA ligase, lysine-tRNA ligase, proline-tRNA ligase, tRNA dimethylallyltransferase, ribosome-recycling factor, and methylenetetrahydrofolate-tRNA-(uracil-5-)-methyltransferase (TrmFO/GidA) and ribosomal maturation factor P (RimP/yhbC) were prominently upregulated. GidA/TrmFO is a multifunctional protein implicated in bacterial virulence, morphology, growth, and antibiotic susceptibility. Deletion mutants in *Salmonella enterica*, *E. coli*, and *Pseudomonas aeruginosa* showed attenuated growth and reduced pathogenicity [36]. Ribosomal maturation factor P (RimP/yhbC) is essential for ribosome assembly, with deletion resulting in lethality in *Streptococcus pneumoniae*, and impaired growth and stress sensitivity in *E. coli* and *Salmonella enteritidis* [37]. Together, these observations indicate that *S. suis* modulates translation machinery to adapt to intracellular environments and maintain virulence.

Carbohydrate metabolism proteins, including galactokinase, phosphoglycerate kinase, 6-phosphofructokinase, and phosphoenolpyruvate carboxylase (PEP), and dTDP-D-glucose 4,6-dehydratase were upregulated in both serotypes. PEP plays a critical role in oxaloacetate synthesis, a key precursor for amino acid and nucleotide metabolism, supporting survival in nutrient-limited host environments such as blood, cerebrospinal fluid, and brain tissues [38,39]. Its conserved upregulation across serotypes—including SS19—highlights metabolic adaptation as a fundamental virulence mechanism [24]. Similarly, dTDP-D-glucose 4,6-dehydratase, involved in nucleotide sugar biosynthesis, influences cell surface glycoproteins and lipopolysaccharides, modulating resistance during exponential growth [39]. These findings align with prior proteomic studies that identified metabolic adaptation as a central strategy for intracellular survival [28,40].

DNA replication proteins, particularly ATP-dependent DNA helicase RuvB, were upregulated. RuvB facilitates branch migration of Holliday junctions and DNA repair, enhancing genetic adaptability and virulence [41,42]. Stress-response proteins, including acetate kinase (AckA) and molecular chaperone DnaK, were also upregulated. DnaK (Hsp70) assists in protein folding under stress and contributes to adhesion and host plasminogen activation, facilitating bacterial dissemination [43]. Prior studies demonstrated that DnaK binds host proteins, promoting intracellular survival and immune evasion in pathogenic bacteria.

The proteomic shifts induced by macrophage contact included significant changes in fundamental bacterial processes such as DNA replication, transcription, translation, and central metabolism. While essential for growth, the high conservation of these pathways across the tree of life presents a formidable challenge for developing selective antimicrobials that avoid host toxicity, a well-documented bottleneck in traditional antibiotic discovery [44].

Modern strategies instead emphasize targeting conditionally essential pathways—those critical for survival within the host environment—and virulence factors, which can offer greater pathogen specificity. This includes stress-response systems, immunogenic surface proteins, nutrient acquisition machinery, and regulatory networks [45].

Comparative analysis revealed that SS2 isolates exhibited a greater number of upregulated proteins than SS14 during intracellular survival. SS2-exclusive proteins included ketol-acid reductoisomerase, phosphate acyltransferase, chorismate synthase, 30S ribosomal proteins S7 and S8, UPF0348 protein SSU98_0368, elongation factor 4 (EF-4), aspartate-tRNA ligase, ATP-dependent helicase/nuclease subunit A, alanine-tRNA ligase, 50S ribosomal protein L1, and UDP-N-acetylglucosamine pyrophosphorylase (GlmU). These proteins have previously been implicated in translational efficiency, growth, and immunogenicity [24,46,47]. GlmU is essential for capsule and peptidoglycan biosynthesis, critical for immune evasion [38]. EF-Tu and EF-G, elongation factors with cytoplasmic and surface-localized functions, support persistence and have shown immunogenicity in SS9 [46,47], suggesting conserved roles across serotypes.

**Figure 9:**
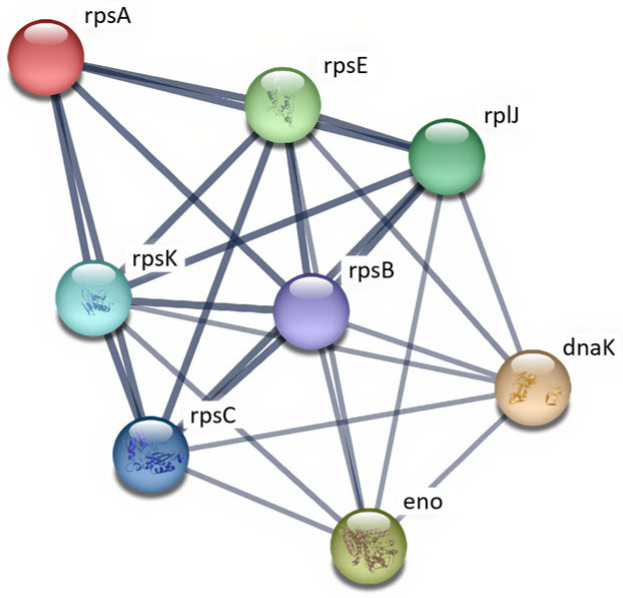
Protein–protein interaction network of enolase associated proteins in *S. suis* SS2 and SS14 isolates from pig during exposure to human macrophages. The network was analyzed by STRING with a confidence score ≥ 0.7. The nodes represent up-regulated proteins. The lines represent the predicted functional associations. The thick lines mean strong interactions between proteins.

For SS14, 30S ribosomal protein S5 was uniquely upregulated in human and pig isolates, potentially impacting translational fidelity, consistent with observations in *E. coli* [35]. Notably, 50S ribosomal protein L33 and enolase were upregulated exclusively in pig-derived isolates. Enolase acts as a virulence factor by binding human plasminogen and fibronectin, inducing antibody production, and disrupting the blood–brain barrier through interaction with 40S ribosomal protein SA, activating p38/ERK-eIF4E signaling and increasing host–cell apoptosis [24,48,49]. Recent studies further demonstrate enolase’s role in co-condensation with host vimentin, Ca^2+^ influx modulation, and enhancement of cytotoxicity in human brain microvascular endothelial cells [50]. These data corroborate our findings and confirm the zoonotic potential of pig-derived isolates. Enolase associated protein interaction are shown in Figure 9.

Despite these insights, this study has limitations. The use of U937 cells may not fully recapitulate the complexity of human immune responses, particularly T-cell-mediated cytokine signaling (e.g., IFN-γ) [51]. Analysis was limited to a single post-invasion time point, which precludes assessment of temporal dynamics in protein expression. Functional roles of identified proteins were inferred from prior literature rather than experimentally validated in human or animal models.

We acknowledge that detergent-only extraction without additional mechanical or enzymatic disruption may underrepresent tightly cell-wall-anchored proteins in Gram-positive bacteria. However, SDS-based solubilization, cold acetone precipitation, differential centrifugation, and in-gel/in-solution digestion are widely used proteomic workflows for intracellular pathogens, and species-specific peptide filtering ensures that host proteins do not enter the bacterial protein dataset.

We employed a standard gentamicin/penicillin protection assay to isolate intracellular bacteria. While this method is established, we did not include a control to assess the potential direct effect of these antibiotics on the *S. suis* proteome. Although the exposure was short and intracellular bacterial viability was confirmed, we cannot exclude that some of the observed proteomic changes, particularly in stress-response pathways, may be influenced by antibiotic exposure [52]. Future studies incorporating antibiotic-exposure controls would help delineate the specific contributions of the intracellular environment versus antibiotic stress.

Complementary to our intracellular proteomic approach, Prados de la Torre et al. (2020) used surface-shaving proteomics and bioinformatic prediction to identify 42 potential vaccine candidates from human *S. suis* isolates, including multifunctional enzymes such as DnaK, enolase, EF-Tu, and GAPDH [53]. These proteins, known for roles in adhesion and immune evasion, were also upregulated during intracellular survival in our macrophage model, supporting their dual functions as metabolic enzymes and “moonlighting” virulence factors. While that study emphasized antigen discovery, our results reveal how these same proteins are dynamically expressed under infection-mimetic intracellular stress. The overlap of DnaK, EF-Tu, and enolase between vaccine-candidate and intracellular proteomes highlights their shared importance in virulence and immunogenicity, underscoring the value of integrating antigenicity prediction, exoproteome profiling, and intracellular proteomics to prioritize multifunctional targets that are both surface-accessible and essential for infection.

Future studies should include time-resolved proteomic analyses to capture dynamic adaptation during infection, validation in primary human macrophages or relevant animal models, and targeted functional mutagenesis or inhibition assays to confirm roles in intracellular survival, virulence, and host–pathogen interactions. Candidate proteins such as PEP, RuvB, EF-4, GlmU, enolase, and DnaK represent promising targets for vaccine or therapeutic development.

## 4. Materials and Methods

### 4.1. Bacterial Strains and Culture Conditions

Five *S. suis* strains were used in this study, including three SS2 strains—P1/7 (reference strain), TSK10.4, and LPH210/53—and two SS14 strains—TD2.2 and MNCM07 (Table 2). Strains originated from diseased pigs, healthy pigs, or human patients as indicated. Bacteria were streaked onto blood agar plates, and single colonies were inoculated into Todd–Hewitt broth (THB; Difco, Detroit, MI, USA). Cultures were grown at 37 °C with 5% CO_2_ to mid-logarithmic phase (3–5 h) and adjusted to an OD_600_ of 0.4 (approximately 10^8^–10^9^ CFU/mL) for all experiments [5].

### 4.2. Cell Cultures

U937 human monocytic cells (leukemic monocyte lymphoma) were maintained in RPMI 1640 medium (Gibco, Grand Island, NY, USA) supplemented with 10% heat-inactivated fetal bovine serum (Gibco, Grand Island, NY, USA), L-glutamine (Gibco, Grand Island, NY, USA), and penicillin–streptomycin. Cells were cultured at 37 °C in 5% CO_2_ and used before the 15th passage. For macrophage differentiation, cells were stimulated with 100 ng/mL phorbol myristate acetate (PMA; Sigma-Aldrich, St. Louis, MO, USA) for 15–18 h before infection [54].

### 4.3. Cell Invasion Assay

The invasion capacity of *S. suis* strains was determined following previously described methods [55,56], with modifications. Mid-log cultures (OD_600_ = 0.4) were centrifuged at 13,000× *g* for 5 min, washed twice with PBS (pH 7.2), and resuspended in antibiotic-free RPMI to 5 × 10^7^ CFU/mL. Differentiated U937 macrophages (~10^6^ cells/well) were infected at MOIs of 1, 10, 50, 100 and 200. The condition was determined to yield optimal invasion efficiency for downstream analyses.

Plates were centrifuged at 2700× *g* for 10 min and incubated for 2 h at 37 °C with 5% CO_2_. Monolayers were washed twice with PBS and treated with gentamicin (100 μg/mL) and penicillin G (10 μg/mL) for 1 h to eliminate extracellular bacteria. This combined antibiotic treatment, standard for *S. suis* intracellular assays, was used for a limited duration (1 h) to minimize potential effects on intracellular bacterial physiology [11,57]. The efficacy of antibiotic treatment was confirmed by plating the final wash on THB agar.

Cells were then incubated with trypsin-EDTA (Gibco, Grand Island, NY, USA) for 10 min, neutralized with FBS, and lysed with sterile distilled water to release intracellular bacteria. Lysates were plated on THB agar and incubated overnight at 37 °C. Invasion efficiency was expressed as CFU recovered per well. Each assay was performed in duplicate and repeated at least three times.

### 4.4. Protein Extraction from Intracellular Bacteria

Intracellular bacteria were harvested by lysing macrophages with 0.1% Triton X-100 in distilled water. Host cell debris was removed by centrifugation at 300× *g* for 10 min at 4 °C. The supernatant, containing bacteria, was centrifuged at 5000× *g* for 10 min to pellet the bacterial cells. This bacterial pellet was washed three times with ice-cold phosphate-buffered saline (PBS; Gibco, Grand Island, NY, USA) to minimize carryover of host proteins. Bacterial lysis was performed by resuspending the pellet in 100 µL of lysis buffer (0.5% SDS, 50 mM TEAB, pH 8.5) and vortexing vigorously for 10 min at room temperature to ensure thorough disruption. The lysate was cleared by centrifugation at 16,000× *g* for 15 min [27,58]. Proteins in the supernatant were precipitated with four volumes of cold acetone overnight at −20 °C. The precipitated protein pellet was air-dried and resuspended in 50 mM TEAB for quantification and subsequent tryptic digestion.

### 4.5. Protein Quantification

Protein pellets were solubilized in 0.15% sodium deoxycholate (DOC), and protein concentrations were determined by the Lowry assay [59]. Absorbance at 750 nm was measured, and concentrations were calculated from a BSA standard curve.

### 4.6. Sodium Dodecyl Sulfate-Polyacrylamide Gel Electrophoresis (SDS-PAGE)

The protein samples were separated by SDS-PAGE mini slab gel (8 × 9 × 0.1 cm, Hoefer miniVE, Amersham Biosciences, UK). The separating gel was prepared according to the standard method described by Laemmli (1970) [60] with a 12.5% polyacrylamide gel. The equal volume of protein samples was mixed with 5 μL of 5X sample buffer (0.125M Tris-HCl pH 6.8, 20% glycerol, 5% SDS, 0.2M DTT, 0.02% bromophenol blue) and boiled at 95 °C for 10 min before loading on the 12.5% SDS-PAGE. Determination of the molecular weight size of proteins was performed by using the standard marker (Amersham Biosciences UK Ltd, Buckinghamshire, UK). Electrophoresis was performed in buffer (25 mM Tris-HCl pH 8.3, 192 mM glycine, 0.1% SDS) until the tracking dye reached the bottom of the gel and then gels were stained with silver staining [61].

### 4.7. In-Solution Digestion

Protein samples were resuspended in 20 µL of 10 mM ammonium bicarbonate, followed by the addition of 5 µL of 10 mM dithiothreitol (DTT) in 10 mM ammonium bicarbonate and incubated at room temperature for one hour. Subsequently, 20 µL of 100 mM iodoacetamide (IAA) in 10 mM ammonium bicarbonate was added, and the mixture was incubated at room temperature for another hour in the dark. Then, 5 µL of trypsin solution (10 ng Trypsin in 50% acetonitrile/10 mM ammonium bicarbonate) was added, and the samples were incubated at room temperature for 1–3 h. The peptide samples were dried using a SpeedVac for 1–2 h then resuspended in 10 to 15 µL of 0.1% trifluoroacetic acid (TFA) for analysis by liquid chromatography–mass spectrometry (LC-MS/MS) [62].

### 4.8. In-Gel Digestion

Protein samples were separated by SDS-PAGE, and ~15 distinct bands were excised into 1 × 1 × 1 mm^3^ gel plugs and transferred to a 96-well plate (5 plugs/well). Gel pieces were washed with 200 µL sterile water, shaken 5 min at room temperature, and dehydrated with 200 µL 100% ACN for 5 min (×3), then dried for 5–10 min. Proteins were reduced with 50 µL 10 mM DTT in 10 mM ammonium bicarbonate for 1 h at room temperature, followed by alkylation with 50 µL 100 mM iodoacetamide in 10 mM ammonium bicarbonate for 1 h in the dark. Gel plugs were dehydrated again with 200 µL ACN (×2) and incubated with 20–40 µL trypsin solution (10 ng trypsin in 50% ACN/10 mM ammonium bicarbonate) for 20 min. Digestion was performed by adding 30 µL 30% ACN and incubating at room temperature for 3 h or overnight. Peptides were extracted with 30 µL 50% ACN in 0.1% formic acid for 10 min, dried at 40 °C for 3–4 h or overnight, pooled into a microcentrifuge tube, and centrifuged at 13,000× *g* for 5 min. Eight microliters of the peptide solution were transferred to LC-MS/MS insert tubes for protein identification [63].

### 4.9. Liquid Chromatography–Mass Spectrometry (LC-MS/MS) Analysis

Peptide digests were analyzed using an HCTultra PTM Discovery System (Bruker Daltonics Ltd., Bremen, Germany) coupled to an UltiMate 3000 LC System ( Dionex UK Ltd, Camberley, UK). Peptides were separated on a PepSwift monolithic nanocolumn (100 μm i.d. × 50 mm). The mobile phase consisted of solvent A (0.1% formic acid in water) and solvent B (80% acetonitrile in water with 0.1% formic acid). Peptide fragment mass spectra were acquired in data-dependent AutoMS(2) mode with a scan range of 300–1500 *m*/*z*, 3 averages, and up to 5 precursor ions selected from the MS scan range of 50–3000 *m*/*z* [64].

### 4.10. Protein Identification and Data Analysis

Protein identification was performed using peptide mass fingerprinting with DeCyder MS Differential Analysis software version 6.0 (DeCyderMS, GE Healthcare, Munich, Germany [65,66]) for protein quantitation. The resulting MS/MS data were submitted for database searching using Mascot software version 2.8.5 (Matrix Science, London, UK [67]). The search parameters were set as follows: enzyme = trypsin, maximum missed cleavages = 1; fixed modification = carbamidomethyl (C); variable modification = oxidation (M); peptide mass tolerance ± 1.2 Da; MS/MS tolerance ± 0.6 Da; peptide charges = 1+, 2+, and 3+; instrument = ESI-TRAP. Proteins were considered significant if they contained more than two peptides with individual Mascot scores corresponding to top < 0.05 and *p* < 0.1. Protein–protein interaction networks were constructed using the STRING database (version 12.0) [68]. The analysis was performed by submitting the UniProt accession numbers of proteins identified as significantly upregulated in our intracellular proteome. The search was restricted to *S. suis* (taxonomy ID: 1307). A high-confidence interaction score threshold of ≥0.700 was applied. All active interaction sources were considered: Textmining, Experiments, Databases, Co-expression, Neighborhood, Gene Fusion, and Co-occurrence. Disconnected nodes were hidden from the resulting network. The network was visualized using the embedded STRING viewer, and the resulting image was exported for figure preparation. These parameters are provided to ensure full reproducibility of the network analysis.

Invasion efficacy of the isolates was compared using two-way ANOVA, and statistical significance was evaluated at *p* = 0.05, *p* = 0.01, and *p* = 0.001. All statistical analyses and generation of histograms were performed using GraphPad Prism version 10.5.0.

## 5. Conclusions

This study provides comprehensive insight into *S. suis* intracellular adaptation under human-relevant conditions, highlighting translation, metabolism, stress response, and DNA repair as conserved mechanisms. Serotype-specific differences reveal multifactorial strategies underlying virulence and zoonotic potential. The integration of these proteomic findings with prior functional studies strengthens the rationale for targeting these pathways in controlling *S. suis* infections in both swine and humans.

## Figures and Tables

**Table 1:** Differentially upregulated proteins identified in *S. suis* SS2 and SS14 isolates during intracellular survival in human U937 macrophages.

Identified Protein	SS2		SS14	
	Patient	Pig	Patient	Pig
Galactokinase	+	+	+	+
6-phosphofructokinase	+	+	+	+
N-acetylmannosamine-6-phosphate 2-epimerase	+	+	+	+
Phosphoenolpyruvate carboxylase	+	+	+	+
Phosphopentomutase	+	+	+	+
Dtdp-D-Glucose 4,6-Dehydratase	+	+	+	+
Adenine phosphoribosyltransferase	+	+	+	+
DNA polymerase III PolC-type	+	+	+	+
DNA-directed RNA polymerase subunit beta	+	+	+	+
Holliday junction ATP-dependent DNA helicase RuvB	+	+	+	+
DNA-directed RNA polymerase subunit omega	+	+	+	+
Holliday junction ATP-dependent DNA helicase RuvA	+	+	+	+
Dihydroxy-acid dehydratase	+	+	+	+
Gamma-glutamyl phosphate reductase	+	+	+	+
GTPase Era	+	+	+	+
Acetate kinase	+	+	+	+
Chaperone protein DnaK	+	+	+	+
competence-damage inducible protein	+	+	+	+
Transcriptional repressor NrdR	+	+	+	+
Polyribonucleotide nucleotidyltransferase	+	+	+	+
Glycine-tRNA ligase beta subunit	+	+	+	+
tRNA dimethylallyl transferase	+	+	+	+
Ribosome-recycling factor	+	+	+	+
Lysine--tRNA ligase	+	+	+	+
UPF0298 protein SSU05_1549	+	+	+	+
Argininosuccinate lyase	+	+	+	+
50S ribosomal protein L18	+	+	+	+
Proline--tRNA ligase	+	+	+	+
30S ribosomal protein S2	+	+	+	+
Glutamate--tRNA ligase	+	+	+	+
UPF0374 protein SSU05_0445	+	+	+	+
Methylenetetrahydrofolate--tRNA-(uracil-5-)-methyltransferase TrmFO	+	+	+	+
Ribosome maturation factor RimP	+	+	+	+
50S ribosomal protein L35	+	+	+	+
50S ribosomal protein L24	+	+	+	+
50S ribosomal protein L5	+	+	+	+
MutS2 protein	+	+	+	+
tRNA uridine 5-carboxymethylaminomethyl modification enzyme MnmG	+	+	+	+
50S ribosomal protein L22	+	+	+	+
30S ribosomal protein S15	+	+	+	+
Peptidyl-tRNA hydrolase	+	+	+	+
30S ribosomal protein S12	+	+	+	+
UDP-N-acetylmuramoyl-L-alanyl-D-glutamate-L-lysine ligase	+	+	+	+
Protein translocase subunit SecA	+	+	+	+
Pyrrolidone-carboxylate peptidase	+	+	+	+
UDP-N-acetylenol pyruvoylglucosamine reductase	+	+	+	+
Ketol-acid reductoisomerase	−	+	−	−
Phosphate acyltransferase	−	+	−	−
Chorismate synthase	−	+	−	−
30S ribosomal protein S7	−	+	−	−
UPF0348 protein SSU98_0368	−	+	−	−
30S ribosomal protein S8	−	+	−	−
Elongation factor 4	−	+	−	−
Aspartate--tRNA ligase	+	−	−	−
ATP-dependent helicase/nuclease subunit A	+	−	−	−
tRNA threonylcarbamoyladenosine biosynthesis protein Gcp	−	−	+	−
Queuine tRNA-ribosyltransferase	−	−	+	−
Purine nucleoside phosphorylase DeoD-type	−	−	+	−
50S ribosomal protein L2	−	−	−	+
Alanine--tRNA ligase	+	+	−	−
50S ribosomal protein L1	+	+	−	−
UDP-N-acetylglucosamine pyrophosphorylase	+	+	−	−
30S ribosomal protein S5	−	−	+	+
50S ribosomal protein L33	−	+	−	+
Enolase	−	+	−	+
Total number	51	58	50	50

+ = Presence of proteins up-regulation. − = Absence of proteins up-regulation.

**Table 2:** Overview of the *S. suis* strains (SS2 and SS14) used for intracellular infection assays and comparative proteomic profiling, with details on strain identity and host origin.

Serotypes	Strains	Specimen	Source of Isolation	Virulence-Associated Genes
2	P1/7 (Reference strain)	Ante-mortem blood culture from a pig dying with meningitis	Diseased pig	*sly*+/*epf*+/*mrp*+
	LPH 210/53	Hemoculture	Patient	*sly*+/*epf*+/*mrp*+
	TSK 10.4	Tonsil	Healthy pig	*sly−*/*epf−*/*mrp*+
14	MNCM 07	Hemoculture	Patient	*sly*+/epf*+/*mrp*+
	TD 2.2	Tonsil	Healthy pig	*sly*+/*epf*+/*mrp*+

+ = Presence of the gene; − = Absence of the gene; epf*, an *epf* variant producing a ~3 kb amplicon as previously described [15].

## Data Availability

The original contributions presented in this study are included in the article and Supplementary Material. For further inquiries, please contact the corresponding author.

## Supplementary Materials

The following supporting information can be downloaded at https://www.mdpi.com/article/10.3390/ijms27010128/s1.

## Abbreviations

The following abbreviations are used in this manuscript:

SDS-PAGE	Sodium dodecyl sulfate–polyacrylamide gel electrophoresis
mM	Millimolar
°C	Degree Celsius
µl	Micriolitter
× *g*	force of gravity; relative centrifugal force (RCF)
p	Probability value, Statistical significance
CFU	Colony Forming Units
min	Minute
ml	Milliliter
μm	Micrometer
mm	Millimeter
h	Hour
i.d.	Internal diameter
*m*/*z*	Mass-to-charge ratio
