# Supplementary material for: Proteomic Analysis of Streptococcus suis During Exposure to Intracellular Condition of Human Macrophage U937 Cells
Source: Int J Mol Sci. 2025 Dec 22;27(1):128. doi: 10.3390/ijms27010128 (PMC12786085; doi:10.3390/ijms27010128)
Supplement: Supplementary file 1 [file ijms-27-00128-s001.zip › ijms-4017662-supplementary.pdf]

**Supplementary**

**Table S1.** Proteins up-regulated in *S. suis* strain TSK 10.4 during exposure to human macrophages

| Protein identification | Peptide sequences | ID score | Function |
|---|---|---|---|
| Ketol-acid reductoisomerase | VGAELRKAMPFVGRNDD DAFK | 3.01 | Carbohydrate metabolism |
| Phosphate acyltransferase | KKEASMVLATK | 0.81 | Lipid metabolism |
| Chorismate synthase | VAVGAVAKR | 4.85 | Protein metabolism |
| 30S ribosomal protein S7 | EIMDAANNTGAAVKK | 8.31 | Translation |
| UPF0348 protein SSU98_0368 | GADQLDLVR | 5.84 | Translation |
| 30S ribosomal protein S8 | RVSKPGLR | 3.7 | Translation |
| Elongation factor 4 | AGKKR | 0.6 | Translation |

* ID score = Identity score

**Table S2.** Proteins up-regulated in *S. suis* strain LPH 210/53 during exposure to human macrophages

| Protein identification | Peptide sequences | ID score | Function |
|---|---|---|---|
| Aspartate--tRNA ligase | AIVVKGAADSYSRK | 4.38 | Translation |
| ATP-dependent helicase/nuclease subunit A | ILDKLKR | 11.16 | DNA replication |

* ID score = Identity score

**Table S3.** Proteins up-regulated in *S. suis* strain MNCM07 during exposure to human macrophages

| Protein identification | Peptide sequences | ID score | Function |
|---|---|---|---|
| tRNA threonylcarbamoyladenosine biosynthesis protein Gcp | VGRVMGLPYPAGR | 5.56 | Translation |
| Queuine tRNA-ribosyltransferase | LTSYHNLYFLINLMK | 2.3 | Translation |
| Purine nucleoside phosphorylase DeoD-type | ILLPGDPLR | 3.13 | DNA metabolism |

* ID score = Identity score

**Table S4.** Proteins up-regulated in *S. suis* strain TD 2.2 during exposure to human macrophages

| Protein identification | Peptide sequences | ID score | Function |
|---|---|---|---|
| 50S ribosomal protein L2 | NKDGVEAIVK | 1.27 | Translation |

* ID score = Identity score

**Table S5.** Proteins up-regulated in all strains of *S. suis* serotype 2 during exposure to human macrophages

| Protein identification | Peptide sequences | ID score | Function |
|---|---|---|---|
| Alanine--tRNA ligase | AIVVKGAADSYSRK | 12.36 | Translation |
| 50S ribosomal protein L1 | SKNLLAALEK | 6.30 | Translation |
| UDP-N-acetylglucosamine pyrophosphorylase | ILDKLKR | 1.00 | DNA replication |

* ID score = Identity score

**Table S6.** Proteins up-regulated in both strains of *S. suis* serotype 14 during exposure to human macrophages

| Protein identification | Peptide sequences | ID score | Function |
|---|---|---|---|
| 30S ribosomal protein S5 | AEEVAALR | 6.93 | Translation |

* ID score = Identity score

**Table S7.** Proteins up-regulated in *S. suis* isolates from pigs both serotype 2 and serotype 14

| Protein identification | Peptide sequences | ID score | Function |
|---|---|---|---|
| 50S ribosomal protein L33 | RNTPDRLQLK | 9.35 | Translation |
| Enolase | GLVTAVGDEGGFAPK | 57.28 | Unknown |

* ID score = Identity score

**Table S8.** Proteins of *S. suis* serotype 2 and serotype 14 that were similarly up-regulated during exposure to human macrophages.

| Protein identification | Peptide sequences | ID score* | Function |
|---|---|---|---|
| Galactokinase | RAECEK | 9.38 | Carbohydrate metabolism |
| 6-phosphofructokinase | TFVVEVMGR | 16.01 | Carbohydrate metabolism |
| N-acetylmannosamine-6-phosphate 2-epimerase | EEGGIMPLLVK | 4.77 | Carbohydrate metabolism |
| Phosphoenolpyruvate carboxylase | RFIDK | 8.04 | Carbohydrate metabolism |
| Phosphopentomutase | IGLGNIPR | 12.05 | Carbohydrate metabolism |
| Dtdp-D-Glucose 4,6-Dehydratase | QITNILAGIKPK | 0.71 | Carbohydrate metabolism |
| Adenine phosphoribosyltransferase | VLMHY | 7.66 | DNA metabolism |
| DNA polymerase III PolC-type | LAAGAAGVKR | 15.59 | DNA replication |
| DNA-directed RNA polymerase subunit beta | EIAALKEELK | 16.16 | DNA replication |
| Holliday junction ATP-dependent DNA helicase RuvB | IFIEAAK | 11.01 | DNA replication |
| DNA-directed RNA polymerase subunit omega | MMLKPSIDTLLDK | 2.06 | DNA replication |
| Holliday junction ATP-dependent DNA helicase RuvA | GILTKITAK | 4.02 | DNA replication |

* ID score = Identity score

**Table S8.** (Continued)

| Protein identification | Peptide sequences | ID score* | Function |
|---|---|---|---|
| Dihydroxy-acid dehydratase | KADIEEAGR | 12.94 | Protein metabolism |
| Gamma-glutamyl phosphate reductase | IDLLVPR | 5.76 | Protein metabolism |
| GTPase Era | GIIIGK | 12.87 | Signal transduction |
| Acetate kinase | EVERLK | 7.63 | Stress response |
| Chaperone protein DnaK | AQALAVK | 11.64 | Stress response |
| competence-damage inducible protein | VRTPNNER | 7.95 | Stress response |
| Transcriptional repressor NrdR | GSKVKPGKK | 14.1 | Transcription |
| Polyribonucleotide nucleotidyltransferase | QANGAVVVR | 6.9 | Transcription |
| Glycine-tRNA ligase beta subunit | VGAVLALADK | 10.56 | Translation |
| tRNA dimethylallyl transferase | QVLYDR | 9.53 | Translation |
| Ribosome-recycling factor | MTADKEKELLEV | 8.35 | Translation |
| Lysine--tRNA ligase | MTALAEQGIDPFGK | 8.15 | Translation |
| UPF0298 protein SSU05_1549 | ATILKLKK | 3.03 | Translation |
| Argininosuccinate lyase | KNPDMAELIR | 11.86 | Translation |
| 50S ribosomal protein L18 | LNIFR | 23.31 | Translation |

* ID score = Identity score

**Table S8.** (Continued)

| Protein identification | Peptide sequences | ID score | Function |
|---|---|---|---|
| Proline--tRNA ligase | AADGIVEVK | 11.37 | Translation |
| 30S ribosomal protein S2 | MEEDGTFEVLPK | 1.81 | Translation |
| Glutamate--tRNA ligase | SIQHIDNMLKSL | 4.44 | Translation |
| UPF0374 protein SSU05_0445 | TWRDTMVLK | 1.29 | Translation |
| Methylenetetrahydrofolate--tRNA-(uracil-5-)-methyltransferase TrmFO | RLDSIIMR | 7.47 | Translation |
| Ribosome maturation factor RimP | KEVTIPYQTVAK | 2.05 | Translation |
| 50S ribosomal protein L35 | RTGSGGLKR | 12.74 | Translation |
| 50S ribosomal protein L24 | GVEAVVVTALPK | 4.36 | Translation |
| 50S ribosomal protein L5 | LVTVSLPR | 11.39 | Translation |
| MutS2 protein | ARLDLR | 21.77 | Translation |
| tRNA uridine 5-carboxymethylaminomethyl modification enzyme MnmG | GLENAQMMR | 7.54 | Translation |
| 50S ribosomal protein L22 | AKGSASPINK | 7.34 | Translation |
| 30S ribosomal protein S15 | NLLAYLRR | 7.2 | Translation |
| Peptidyl-tRNA hydrolase | LIIGLGNPGDR | 5.66 | Translation |
| 30S ribosomal protein S12 | SPALNVGYNSRKK | 4.2 | Translation |
| UDP-N-acetylmuramoyl-L-alanyl-D-glutamate-L-lysine ligase | LLAFTGTKGK | 2.16 | Transport |
| Protein translocase subunit SecA | DEAIDGIIK | 5.73 | Transport |
| Pyrrolidone-carboxylate peptidase | AMVEAIHR | 3.23 | Transport |
| UDP-N-acetylenol pyruvoylglucosamine reductase | MKDKIR | 1.19 | Transport |

* ID score = Identity score

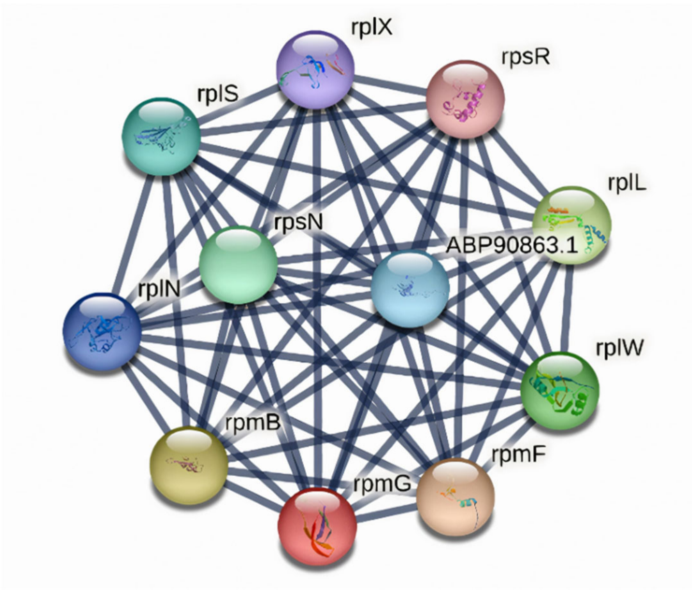


**Figure S1.** Protein–protein interaction network of protein up regulated in isolate from patient

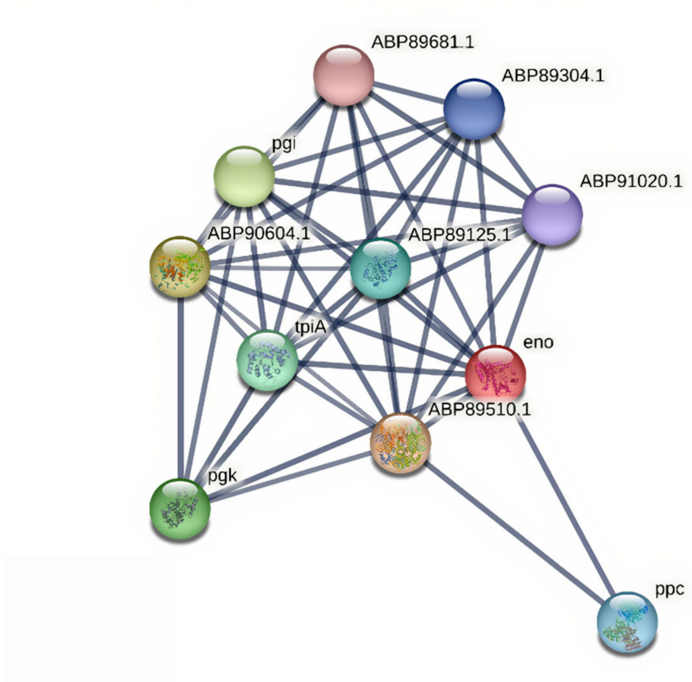


**Figure S2.** Protein–protein interaction network of protein up regulated in isolate from pig
